# Topical TRPM8 Agonist for Relieving Neuropathic Ocular Pain in Patients with Dry Eye: A Pilot Study

**DOI:** 10.3390/jcm10020250

**Published:** 2021-01-12

**Authors:** Hyeon Jeong Yoon, Jonghwa Kim, Jee Myung Yang, Edward T. Wei, Seong Jin Kim, Kyung Chul Yoon

**Affiliations:** 1 Department of Ophthalmology, Chonnam National University Medical School and Hospital, 42 Jebong-ro, Dong-gu, Gwangju 61469, Korea; yoonhyeonjeong@hanmail.net (H.J.Y.); ccaaacc@hanmail.net (J.K.); 2 Department of Ophthalmology, Asan Medical Center, University of Ulsan College of Medicine, 88, Olympic-Ro 43 Gil, Songpa-gu, Seoul 05505, Korea; jeemang87@gmail.com; 3 School of Public Health, University of California, Berkeley, CA 94720, USA; koolicin@yahoo.com; 4 Department of Dermatology, Chonnam National University Medical School and Hospital, 42 Jebong-ro, Dong-gu, Gwangju 61469, Korea

**Keywords:** TRPM8 agonist, cryosim-3, dry eye, neuropathic pain

## Abstract

Background: Activation of TRPM8, a cold-sensing receptor located on the cornea and eyelid, has the potential to relieve the neuropathic ocular pain (NOP) in dry eye (DE) by inhibiting other aberrant nociceptive inputs. We aimed to investigate the effect of a topical TRPM8 agonist, cryosim-3 (C3), on relieving DE-associated NOP. Methods: We conducted a prospective pilot study of 15 patients with DE-associated NOP. These patients applied topical C3 to their eyelid, 4 times/day for 1 month. The patients underwent clinical examinations. They also completed the Ocular Pain Assessment Survey (OPAS), which is a validated questionnaire for NOP, at baseline, 1 week, and 1 month after treatment. Result: At 1 week, the OPAS scores of eye pain intensity, quality of life (driving/watching TV, general activity, sleep, and enjoying life/relations with other people), and associated factors (burning sensation, light sensitivity, and tearing) improved. The total OPAS scores of eye pain intensity, quality of life, and associated factors remained improved at 1 month. The Schirmer test scores also improved at 1 month. Conclusion: TRPM8 agonist (C3) could be a novel agent for treating patients with DE-associated NOP who are unresponsive to conventional treatments.

## 1. Introduction

Dry eye (DE) is a multifactorial disease of the ocular surface characterized by a loss of homeostasis of the tear film and accompanying ocular symptoms [1]. It has a prevalence of 10% to 70% [1]. Some patients with DE experience severe pain that reduces their quality of life (QoL) with minimal ocular signs [1]. Topical agents could be applied as a part of DE treatment to reduce inflammation and tear film osmolality [2]. Generally, if the ocular pain cannot be resolved with topical treatment, other specific causes should be suspected, in particular, neuropathic pain could be the underlying cause [3,4]. In DE, ocular pain disproportionally outweighing the clinical signs is suggestive of underlying neuropathic ocular pain (NOP) nature [4].

Transient receptor potential (TRP) cation channels are associated with the perception of chemical and temperature stimulations [5]. Within the TRP family, TRPM8 is a cold-sensing receptor located on nerve endings of the ophthalmic branch of the trigeminal nerve [6]. Since the activation of TRPM8 can inhibit other aberrant nociceptive inputs, agents for targeting this channel might have the potential to relieve the NOP in DE [7,8]. In particular, TRPM8 is distributed in not only cornea but also eyelid; therefore, it can be activated using topical agents that are applied onto the eyelid without directly instilling eye drops to the cornea [6,9,10]. In our previous study, we revealed the effectiveness of topical cryosim-3 (C3)—a water-soluble and selective TRPM8 agonist—in the treatment of DE by increasing basal tear secretion and alleviating ocular discomfort without any complications [9]. In this pilot study, we aimed to investigate the effect of the topical TRPM8 agonist (C3) on relieving NOP in patients with DE.

## 2. Methods

This prospective nonrandomized pilot study was conducted in accordance with the tenets of the Declaration of Helsinki. Ethical approval was obtained from the Chonnam National University Hospital Institutional Review Board (CNUH-2018-274). Informed consent was obtained from all included patients. The sample size was calculated using the G*Power software (version 3.1.9.4; Heinrich-Heine University, Germany) with a level of α = 0.05 and a power of 95% to detect a 2-point difference in pain scales. Accordingly, a total sample size of 13 patients was found sufficient.

Patients with DE accompanied by NOP features, who underwent evaluation between January and December in 2018, were enrolled. DE was diagnosed based on ocular surface disease index (OSDI) score ≥13 and tear break-up time (TBUT) ≤7 s. The inclusion criteria were as follows: (1) chronic ocular pain that was unresponsive to conventional topical agents (i.e., lubricants, anti-inflammatories, and secretagogues) for >3 months; (2) discordance between the painful DE symptoms and signs and specific descriptors, including burning or stinging; and (3) a Wong–Baker FACES Pain Rating Scale (WBFPS) score ≥4. Patients who had a history of ocular diseases other than DE and those receiving systemic medications that alter the pain and mood statuses were excluded.

**Figure 1:**
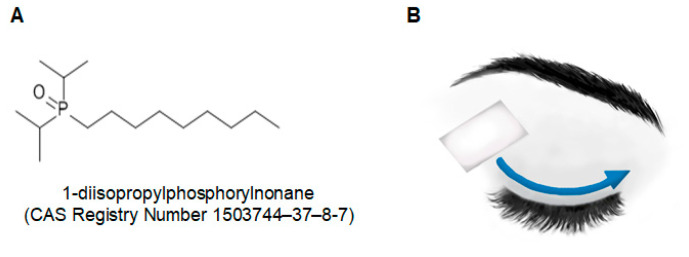
Chemical structure of cryosim-3 (**A**) and the method of topical application of the gauze containing cryosim-3, which targets TRPM8 on the eyelid margin (**B**).

The patients were treated with add-on C3 while undergoing conventional topical treatment. C3 samples (2 mg/mL) were diluted in purified water, soaked in gauze, and packaged using automated equipment. The patients applied topical C3 by wiping the gauze on the closed eyelid margin, 4 times/day for 1 month (Figure 1B).

The OSDI questionnaire, which ranged from 0 to 100, was used to quantify the vision-related QoL. TBUT, the time interval between the last complete blink and the first appearance of disruption of the tear film, was measured thrice, and the mean value was used for analysis. Corneal staining scores were assessed using the area-density index, by multiplying the area and density score. The Schirmer test score represented the length of wetting, and was measured using a calibrated sterile strip placed at the lateral canthus for 5 min under topical anesthesia (0.5% proparacaine). Only the score of the right eye was assessed.

The WBFPS was chosen to screen the pain severity in the patients with DE. The patients chose the face that best depicted the pain they were experiencing. At baseline, 1 week, and 1 month after treatment, the patients also completed the OPAS, which is a validated questionnaire for neuropathic pain as previously described [11]. The questions were divided into sections for analysis: questions 4–9 pertained to eye pain intensity (0 to 60); questions 10–11 pertained to non-eye pain (0 to 20); questions 13–19 (0–10, total score 0 to 60) assessed the QoL (reading and/or computer use, driving and/or watching TV, general activity, mood, sleep, and enjoying life/relations with other people); questions 20–21 (each score 0–1, total score 0–2) assessed aggravating factors (mechanical and chemical stimuli); questions 22–25 (each score 0–1, total score 0–4) assessed associated factors (redness, burning, sensitivity to light, and tearing). The section on symptomatic relief of the OPAS was excluded, and only questions 4–25 were analyzed. The questions were divided into 5 sections as follows: eye pain intensity, non-eye pain, QoL, aggravating factors, and associated factors.

Statistical analyses were conducted using PASW Statistics for Windows, Version 18.0 (SPSS Inc., Chicago, IL, USA). The normality of distribution was assessed using the Shapiro–Wilk test. The Wilcoxon signed-rank test and repeated-measures analysis of variance with Bonferroni’s post hoc test were used for comparing parameters before and after treatment. A *p-*value <0.05 was considered statistically significant.

## 3. Results

This study enrolled 20 patients with DE accompanying NOP features. Five patients (25.0%) discontinued the treatment because of drug ineffectiveness or intolerance. The remaining 15 patients (75.0%) were included in the analysis. Their mean age was 59.5 ± 13.0 years, and nine patients (60.0%) were women. Five patients had a history of intraocular surgery, and one patient had a history of ocular trauma. 

At 1 week after treatment, eye pain intensity, QoL (driving/watching TV, general activity, sleep, and enjoying life/relations with other people), and associated factors (burning sensation, light sensitivity, and tearing) were improved. The total Ocular Pain Assessment Survey (OPAS) scores of eye pain intensity, QoL (sleep), and associated factors (burning sensation and light sensitivity) remained improved at 1 month. However, the score of non-eye pain and aggravating factors did not change after treatment (Table 1). Among the clinical DE parameters, OSDI and Schirmer test score were improved at 1 month after treatment (Table 2). There were no significant differences in pain scores according to previous medications (Supplementary Table S1).

## 4. Discussion

DE is a multifactorial disease of the ocular surface that is accompanied by ocular symptoms [1]. The prevalence of DE has increased considerably worldwide over the last three decades [1]. Some patients with DE experience ocular pain that affects their QoL without any specific abnormal ocular signs [1]. The classification of pain is based on the underlying etiology: (1) nociceptive pain caused by actual or threatened damage to tissues due to the activation of nociceptors, and (2) neuropathic pain caused by a lesion or disease of the somatosensory nervous system [12]. Repeated peripheral nerve injury can lead to peripheral sensitization, and prolonged peripheral ectopic pain initiates central sensitization [4]. Ocular pain symptoms disproportionally outweighing the clinical signs are suggestive of an underlying NOP that might require specific management including systemic treatment [4]. 

However, chronic NOP associated with DE is a challenging clinical problem that is difficult to treat with conventional medications [4,13]. Conventional topical agents such as cyclosporine A could decrease the release of proinflammatory neuropeptides and cytokines from injured nerves, thereby affecting nociceptive pain and peripheral sensitization [13]. However, these topical treatments appear to have limitations in producing an improvement in the corneal nerve morphologic status and central sensitization in patients with chronic NOP. Current systemic medication mainly includes oral antidepressants, anticonvulsants, or gabapentinoid; however, these systemic treatments have several limitations, such as delayed onset, variable efficacy, and unacceptable side effects [4,13,14]. In addition, limited data are available to support the use of systemic neuropathic pain medications for NOP associated with DE [14,15,16]. In this regard, topical agents that are rapid acting, effective, and safe are needed for treating the NOP in DE.

Several members of the TRP super family have emerged as important targets for pain control owing to their critical role in nociception, especially, in chronic states [5]. TRP receptors have been identified in the cornea (TRPV1-4, TRPA1, TRPC4, and TRPM8), conjunctiva (TRPV1, TRPV2, and TRPV4), and eyelid (TRPM8) [6]. In addition, many studies have reported an association between the dysfunction of TRP channels and DE [3,6,17]. TRPM8 is the principal receptor associated with sensing coolness and regulates lacrimal function via response to evaporative cooling and hyperosmolar stimuli [10,18,19,20]. Several studies have showed that cooling the periocular area with an ice pack or instilling cold artificial tears into the eye could relieve ocular pain after surgery [21,22]. Both TRPM8 agonists and antagonists are considered therapeutic agents for pain control [5,6,7,23]. TRPM8 antagonists were shown to improve acute and chronic pain such as cold allodynia [23,24]. However, TRPM8 antagonists can reduce basal tear secretion as an undesirable side effect in DE, as shown in the result of experiments using TRPM8 knock-out mice [20]. TRPM8 agonist could present significant anti-allodynic activity through an excessive activation of TRPM8, leading to its downregulation [25]. Furthermore, TRPM8 agonists have been found to have analgesic effects on neuropathic pain, such as chemotherapy-induced neuropathic pain [8,26,27].

**Figure 2:**
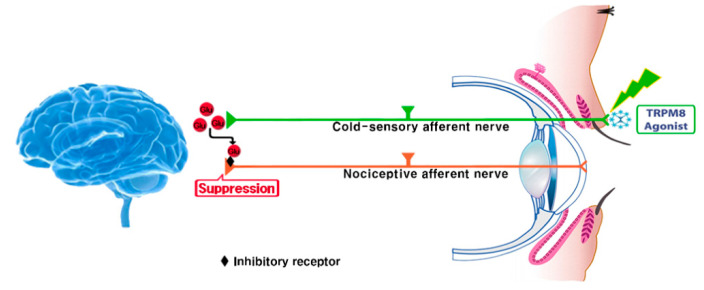
Schematic illustrating the mechanism of action of the TRPM8 agonist in relieving ocular pain in patients with dry eye.

This pilot study showed that the topical application of a TRPM8 agonist (C3) to the eyelid was safe and effective in relieving NOP in patients with DE. We previously showed that the topical application of C3 stimulates basal tear secretion and relieves ocular discomfort in patients with mild DE [9]. The sensory fibers of TRPM8, which innervate the upper eyelid and cornea, are located in the ophthalmic branch of the trigeminal nerve [6]. We speculated in this study that TRPM8 signaling via the eyelid margins may be perceived in the brain as signals from not only the cornea but also the entire ocular surface [9]. Activation of TRPM8 leads to the central synaptic release of glutamate, which then suppresses the injury-activated nociceptive afferent neurotransmission through inhibitory receptors at nerves ending (Figure 2) [8]. In addition, a hypothesis suggests that these actions attenuate neuropathic sensitization on the dorsal horn [8]. In addition, OSDI and Schirmer test scores improved, but TBUT and corneal staining scores remained unchanged after C3 treatment. TRPM8 agonist is known to increase the basal tear secretion and reduce ocular discomfort via neuronal action, but it does not have direct effect on the tear film [6,9]. These results were consistent with our previous study [9].

Topical delivery of C3 to the eyelid margins could minimize corneal exposure that induces side effects, such as discomfort or paradoxical ocular pain [9]. In addition, the wiping of C3 was more comfortable for patients than conventional instillation of eye drops, and produced a painless cooling sensation lasting approximately 40 min [9]. The OPAS scores also decreased at 1 week after treatment, indicating that the topical drug produces effect faster than systemic drugs do [14]. Moreover, although the effect was temporary, C3 was particularly effective when the patients experienced severe pain due to DE, such as when driving or sleeping, thereby resulting in an improved QoL.

This study included a short follow-up period of 1 month and a small sample size. In our study, 15 patients who were included in the analysis showed improved symptoms after treatment; however, a larger sample size would have yielded a more accurate response rate. The number of enrolled patients was too small to perform a subgroup analysis. This was a single-center study, and hence, the findings should be verified in future multicenter prospective randomized control studies evaluating the objective signs. 

In addition, we did not strictly control for previous medications for DE when enrolling the patients. This might have induced a bias during analysis. However, patients in our study did not respond to conventional treatment for a long period of time (122.7 days), but they showed an improvement of ocular pain within 1 week after C3 treatment. This improvement suggests a direct effect of C3 treatment rather than a delayed effect of previous conventional treatment. We believe that including patients with varying histories of medical treatments may likely emulate the actual use of this drug. Despite the aforementioned limitations, the TRPM8 agonist (C3) could be a novel agent for treating NOP in patients with DE who are unresponsive to conventional topical treatment. 

## Figures and Tables

**Table 1:** Changes in the Ocular Pain Assessment Survey scores after the application of cryosim-3 for 1 month.

	Baseline ^a^	1 Week ^b^	1 Month ^c^	*p*-Value *		
				a vs. b	a vs. c	b vs. c
Eye pain intensity (0–60)	30.60 ± 12.84	26.47 ± 11.45	21.53 ± 10.84	0.009	0.015	0.073
Non-eye pain (0–20)	7.67 ± 6.22	6.73 ± 6.18	5.47 ± 5.62	0.999	0.435	0.409
Quality of life (total 0–60)	33.53 ± 14.24	27.60 ± 15.49	27.17 ± 16.06	0.003	0.022	0.743
Reading and/or computer use (0–10)	7.79 ± 1.76	7.14 ± 2.48	6.93 ± 2.59	0.120	0.054	0.272
Driving and/or watching TV (0–10)	6.80 ± 2.31	5.27 ± 2.52	5.60 ± 2.90	0.002	0.070	0.417
General activity (walking, etc.) (0–10)	4.00 ± 3.18	3.27 ± 2.71	3.20 ± 2.86	0.016	0.138	0.843
Mood (0–10)	5.40 ± 2.77	4.53 ± 2.50	4.40 ± 2.47	0.121	0.177	0.769
Sleep (0–10)	4.27 ± 3.81	2.93 ± 3.67	2.73 ± 3.81	0.027	0.049	0.486
Enjoying life/relations with other people (0–10)	5.07 ± 2.84	4.33 ± 2.97	4.27 ± 3.03	0.036	0.068	0.806
Aggravating factors (total 0–2)	1.11 ± 0.49	0.87 ± 0.56	0.88 ± 0.57	0.113	0.132	0.077
Mechanical stimuli (0–1)	0.63 ± 0.29	0.47 ± 0.25	0.47 ± 0.26	0.068	0.086	0.999
Chemical stimuli (0–1)	0.47 ± 0.35	0.41 ± 0.35	0.41 ± 0.32	0.363	0.432	0.872
Associated factors (total 0–4)	2.09 ± 0.76	1.55 ± 0.85	1.58 ± 0.93	0.006	0.046	0.835
Redness (0–1)	0.41 ± 0.32	0.41 ± 0.30	0.39 ± 0.30	0.094	0.104	0.080
Burning sensation (0–1)	0.57 ± 0.37	0.40 ± 0.33	0.29 ± 0.29	0.007	0.002	0.015
Sensitivity to light (0–1)	0.76 ± 0.24	0.57 ± 0.26	0.59 ± 0.28	0.005	0.030	0.663
Tearing (0–1)	0.36 ± 0.29	0.17 ± 0.18	0.21 ± 0.27	0.013	0.197	0.578

All values are presented as mean ± SD. * Compared using repeated measures analysis of variance with Bonferroni’s post hoc test. (a) means “baseline”, (b) means “1-week”, and (c) means “1-month”

**Table 2:** Changes in clinical parameters after the application of cryosim-3 for 1 month.

	BASELINE	1 Month	Z	*p*-Value
Ocular surface disease index	57.5 ± 13.8	40.2 ± 12.6	−3.41	0.001
Tear break-up time (s)	4.13 ± 0.83	4.00 ± 0.85	−0.82	0.414
Schirmer test score (mm)	7.07± 2.76	8.47 ± 2.80	−3.02	0.003
Corneal staining score (0–9)	0.60 ± 0.91	0.13 ± 0.35	−1.82	0.068

All values are presented as mean ± SD. Compared using the Wilcoxon signed rank test.

## Data Availability

The data presented in this study are available on request from the corresponding author. The data are not publicly available due to ethical issue.

## Supplementary Materials

The following are available online at https://www.mdpi.com/2077-0383/10/2/250/s1, Table S1: Previous topical treatment and Wong-Baker FACES Pain Rating Scale (WBFPS) score in enrolled patients.
