# Supplementary material for: Topical TRPM8 Agonist for Relieving Neuropathic Ocular Pain in Patients with Dry Eye: A Pilot Study
Source: J Clin Med. 2021 Jan 12;10(2):250. doi: 10.3390/jcm10020250 (PMC7826705; doi:10.3390/jcm10020250)
Supplement: Supplementary file 1 [file jcm-10-00250-s001.pdf]

Supplementary Table 1. Previous topical treatment and Wong-Baker FACES Pain Rating Scale (WBFPS) score in enrolled patients

Patient No.	Previous treatment		WBFPS score		
	Topical agents	Period (days)	Baseline	1 week	1 month
1	HA 0.1%, CsA 0.05%, diquafosol	171	9	6	4
2	HA 0.15%	95	4	1	2
3	HA 0.15%, 0.1% flumetholone, diquafosol	101	7	7	5
4	HA 0.15%, 0.1% flumetholone, diquafosol	108	7	6	6
5	HA 0.15%, diquafosol	142	6	3	3
6	HA 0.15%, carbomer, 0.1% flumetholone, diquafosol	92	6	5	5
7	HA 0.1%, CsA 0.05%, 0.5% loteprednol, diquafosol	99	6	6	6
8	HA 0.3%, carbomer	155	8	5	4
9	HA 0.3%, diquafosol	144	6	6	5
10	HA 0.3% [A], carbomer [A], diquafosol	135	8	6	4
11	HA 0.15%, CsA 0.05%, 0.1% flumetholone, diquafosol	117	4	1	1
12	HA 0.3%, CsA 0.05%, 0.1% flumetholone	106	8	7	7
13	HA 0.15%, 0.1% flumetholone, diquafosol	102	7	7	5
14	HA 0.18%, CsA 0.05%, diquafosol	181	4	1	1
15	HA 0.3%, carbomer, CsA 0.05%, 0.5% loteprednol	93	4	1	1
		122.7 ± 28.7	6.27 ± 1.61	4.53 ± 2.33	3.93 ± 1.88

Mann-Whitney U analysis	Baseline	1 week	1 month
Previous CsA (yes/no)	0.607	0.456	0.456
Previous Steroid (yes/no)	0.694	0.281	0.121
Previous secretagogues (yes/no)	0.928	0.516	0.710

HA, hyaluronic acid; CsA, cyclosporin A
